# Hydrogen Sulfide: A Gaseous Mediator and Its Key Role in Programmed Cell Death, Oxidative Stress, Inflammation and Pulmonary Disease

**DOI:** 10.3390/antiox11112162

**Published:** 2022-10-31

**Authors:** Zhixing Zhu, Xihua Lian, Madhav Bhatia

**Affiliations:** 1 Department of Pathology and Biomedical Science, University of Otago, Christchurch 8140, New Zealand; 2 Department of Internal Medicine (Pulmonary and Critical Care Medicine), The Second Clinical Medical School of Fujian Medical University, Quanzhou 362002, China

**Keywords:** hydrogen sulfide, gaseous mediator, metabolism, programmed cell death, oxidative stress, inflammation, pulmonary diseases, therapeutic target

## Abstract

Hydrogen sulfide (H_2_S) has been acknowledged as a novel gaseous mediator. The metabolism of H_2_S in mammals is tightly controlled and is mainly achieved by many physiological reactions catalyzed by a suite of enzymes. Although the precise actions of H_2_S in regulating programmed cell death, oxidative stress and inflammation are yet to be fully understood, it is becoming increasingly clear that H_2_S is extensively involved in these crucial processes. Since programmed cell death, oxidative stress and inflammation have been demonstrated as three important mechanisms participating in the pathogenesis of various pulmonary diseases, it can be inferred that aberrant H_2_S metabolism also functions as a critical contributor to pulmonary diseases, which has also been extensively investigated. In the meantime, substantial attention has been paid to developing therapeutic approaches targeting H_2_S for pulmonary diseases. In this review, we summarize the cutting-edge knowledge on the metabolism of H_2_S and the relevance of H_2_S to programmed cell death, oxidative stress and inflammation. We also provide an update on the crucial roles played by H_2_S in the pathogenesis of several pulmonary diseases. Finally, we discuss the perspective on targeting H_2_S metabolism in the treatment of pulmonary diseases.

## 1. Introduction

Pulmonary diseases, including acute respiratory disorders such as acute lung injury (ALI)/acute respiratory distress syndrome (ARDS) and pneumonia, as well as chronic respiratory disorders, such as chronic obstructive pulmonary disease (COPD), asthma and lung cancer, are the leading causes of morbidity, mortality and disability worldwide. Worse still, the absolute numbers of cases of pulmonary diseases have increased globally over the past three decades [1,2,3,4]. The pathogenesis of pulmonary diseases is complex and not yet fully understood. However, dysregulated programmed cell death, increased oxidative stress and aberrant inflammation have attracted substantial attention as they are extensively involved in the development and progression of a plethora of pulmonary diseases [5,6,7].

Well-regulated programmed cell death is of essence in controlling homeostatic functions since eliminating redundant, severely injured, infected and potentially detrimental cells are crucial to cell turnover as well as cellular and organismal homeostasis [8,9,10]. While excessive cell death can cause tissue injury and destruction, a lack of cell death is also involved in many pathological conditions [10]. Notably, dysregulation of programmed cell death in multiple forms, ranging from apoptosis to necroptosis, has been implicated in many pulmonary diseases [5]. In addition, countermeasures targeting the pathways involved in programmed cell death have shown its therapeutic promise in many pulmonary diseases [5,11,12].

The maintenance of redox homeostasis greatly relies on the competent endogenous antioxidant defenses within our bodies. As a result, when endogenous antioxidant defenses fail, the redox homeostasis can be disrupted and further result in an increase in cellular oxidative stress [13,14]. Since the balance between the generation and the clearance of free radicals is disrupted, the redox signaling is disturbed and functions of tissues and organs are affected [15]. Increased oxidative stress has been observed in various diseases, especially pulmonary diseases [6]. Likewise, antioxidant-based therapeutic strategies have been developed and applied in the management of many pulmonary diseases [6,16,17].

Inflammation is a natural process driven by the immune system in response to infection and injury. Inflammation serves as an indispensable mechanism to re-establish homeostasis in cells and tissues in the face of harmful stimuli. Therefore, an immediate, effective and tightly controlled inflammation is a protective reaction that allows us to survive under detrimental conditions [18]. Uncontrolled and unresolved inflammation, on the other hand, can lead to several disorders [19,20]. Of note, aberrant inflammation has been extensively involved in many pulmonary diseases [7]. Similarly, treatments aimed at resolving inflammation function as a key contributor to the re-establishment of organismal homeostasis in many pulmonary diseases [6,7,21,22].

Hydrogen sulfide (H_2_S) has emerged as a key gaseous regulator participating in programmed cell death, oxidative stress and inflammation. Considering the significant roles played by dysregulated cell death, increased oxidative stress and aberrant inflammation in the pathogenesis of pulmonary diseases [5,6,7], it can be inferred that H_2_S is able to affect the pathophysiology of pulmonary diseases as well. H_2_S is extensively involved in the physiopathology of the airways and dysregulated H_2_S metabolism is linked to the onset, development and progression of a plethora of pulmonary diseases [23,24,25]. In addition, targeting H_2_S metabolism presents a novel alternative therapeutic approach against pulmonary diseases, which has aroused significant interest and is being extensively investigated [23,24,25].

In this review, we aim to provide an update on the pathways involved in the metabolism of H_2_S. In addition, we seek to summarize the cutting-edge knowledge on the roles of H_2_S in the pathogenesis of pulmonary diseases. We also attempt to provide new insights to consider interfering with H_2_S metabolism as a therapeutic approach for pulmonary diseases.

## 2. Overview of H_2_S Metabolism and H_2_S Research Tools

H_2_S is a colorless and flammable gas and has a distinctive odor of rotten eggs. Similar to nitric oxide and carbon monoxide, H_2_S has been recognized as a gaseous mediator [26,27]. While H_2_S is toxic in moderate to high amounts, it plays a critical role in many cellular functions and biological processes at low concentrations [26,27].

**Figure 1:**
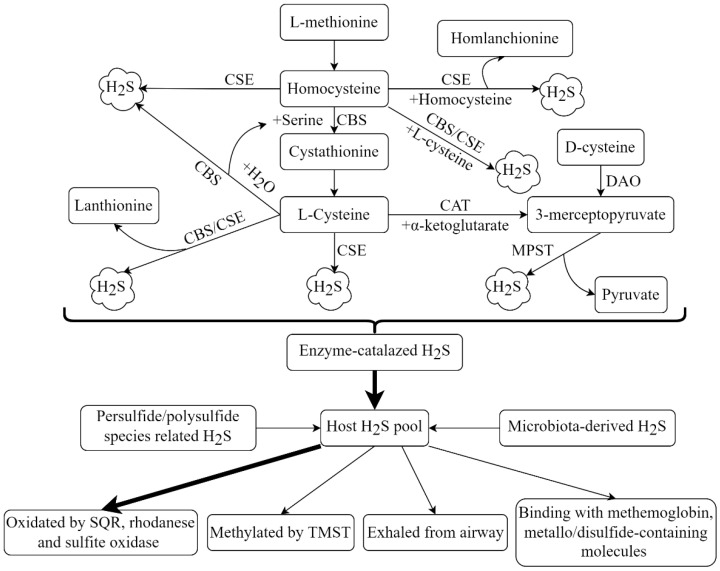
Overview of hydrogen sulfide (H_2_S) metabolism in mammals. Endogenous H_2_S is mainly produced by the desulphydration of L-cysteine or homocysteine by the enzymatic reactions catalyzed by cystathionine β-synthase (CBS), cystathionine γ-lyase (CSE), 3-mercaptopyruvate sulfurtransferase (MPST)/cysteine aminotransferase (CAT) and MPST/D-amino acid oxidase (DAO). Endogenous H_2_S is also yielded from the natural release of H_2_S from persulfide/polysulfide species. Sulfate-reducing microorganism-derived H_2_S is also an important source of endogenous H_2_S. The elimination of endogenous H_2_S is mainly achieved by oxidation in the mitochondria by sulfide quinone oxidoreductase (SQR), rhodanese and sulfite oxidase (SO) and methylation in the cytoplasm by thiol-*S*-methyltransferase (TMST). H_2_S can be directly exhaled from the airways or excreted with biological fluids, such as urine.

The endogenous synthesis of H_2_S in living organisms predominantly arises from the desulphydration of L-cysteine or homocysteine. This conventional source of H_2_S generation accounts for several reactions catalyzed by a group of enzymes, including cystathionine β-synthase (CBS), cystathionine γ-lyase (CSE) and 3-mercaptopyruvate sulfurtransferase (MPST) [28]. Specifically, CBS and CSE, two pyridoxal 5′-phosphate-dependent enzymes, are responsible for transsulfuration reactions, whereas MPST, along with cysteine aminotransferase, catalyze the catabolism of L-cysteine. More recently, it has been proposed that the combined action of MPST and D-amino acid oxidase also contributes to H_2_S biosynthesis [29]. In addition to these enzyme-catalyzed reactions, many other sources, such as the natural release of H_2_S from persulfide and polysulfide species and the yielding of H_2_S from microbiota (sulfate-reducing microorganisms), in the gastrointestinal tract and oral cavity are known [30]. The scavenge of H_2_S in eukaryotic cells involves both enzymatic and nonenzymatic mechanisms. The methylation of H_2_S to methanethiol by thiol-*S*-methyltransferase in the cytoplasm and the oxidation of H_2_S to thiosulfate and sulfate by sulfide quinone oxidoreductase, rhodanese and sulfite oxidase in the mitochondria are the major enzymatic pathways involved in the inactivation of H_2_S [31,32,33]. In addition, H_2_S can be directly exhaled from the airways or excreted from the kidney by binding with methemoglobin and metallo- or disulfide-containing molecules [27,34]. It is worth mentioning that polysulfide species might be involved in the biological and pathological effects of H_2_S as polysulfide species not only act as a natural source of H_2_S production, but also function as a major way to contribute to the elimination of H_2_S [35]. The ways of H_2_S metabolism in mammals are shown in Figure 1.

The understanding of the pathways involved in H_2_S metabolism has given rise to the development of valuable H_2_S research tools, such as H_2_S-producing enzyme inhibitors and H_2_S donor compounds [36,37,38]. These pharmacological inhibitors decrease the host-derived H_2_S, and these donor compounds act as a major source of exogenous H_2_S supplement in research. The development of chemical H_2_S research tools has enhanced our understanding of the role played by H_2_S in many diseases and is comprehensively discussed in several reviews [36,37,38]. The main characteristics of H_2_S-producing enzyme inhibitors and H_2_S donor compounds are briefly summarized in Table 1 and Table 2, respectively. In addition to inhibitors that can suppress the enzymatic activity of H_2_S-synthesizing enzymes, animals with gene deficiency in CBS (heterozygous knockout), CSE and MPST have also been established. These gene-modified animals serve as precise genetic tools in H_2_S-related research and are widely used. Moreover, the development of small interfering RNA targeting CBS, CSE and MPST also contributes to H_2_S-related research.

## 3. Cross-Linking of H_2_S with Cell Death, Oxidative Stress and Inflammation

Many cellular components, that act as damage-associated molecular patterns, are released when death occurs in parenchymal cells. Subsequently, the generation of reactive oxygen species (ROS) is increased and further leads to oxidative stress and inflammation [39,40,41,42]. Under pathological conditions, cell death, oxidative stress and inflammation can lead to each other, thus driving a local auto-amplification loop and further exaggerated cell death, oxidative stress and inflammation in a vicious cycle [43,44,45]. In the past few years, the multifaceted roles of H_2_S as a gaseous regulator in cell death, oxidative stress and inflammation have been widely investigated.

Cell death pathways are extensively involved in the pathogenesis of disorders in many organs and systems [10,11]. As overviewed in some reviews, various forms of cell death have been identified recently and the significant role of H_2_S in regulating cell death in pathological conditions has been discovered [46,47,48,49]. Autophagy is a crucial molecular mechanism for controlling homeostasis in cells and tissues [46,50]. The actions of H_2_S in autophagy are controversial since H_2_S exerts both pro-autophagy and anti-autophagy effects on autophagy [47]. Canonical pyroptosis occurs when the nucleotide-binding oligomerization domain-like receptor protein 3 (NLRP3) inflammasome is activated by inflammatory assault [46,51]. The role of H_2_S in pyroptosis has become a research hotspot. On the one hand, H_2_S effectively inhibited the activation of NLRP3 inflammasome, by which H_2_S provided protection against many pathological conditions, such as liver injury, kidney injury, stroke, diabetes, lung injury, colitis and endothelial dysfunction [48]. On the other hand, H_2_S has also been proposed to promote disease development by activating NLRP3 inflammasome [48]. Probably, the opposite influences of H_2_S on autophagy and NLRP3 inflammasome activation observed among different investigations are due to different doses, timeframes and reaction times of the H_2_S used, and H_2_S may also function distinctively among different organs and systems and different diseases or different disease stages [47,48]. Similarly, there is no general agreement regarding the effects of H_2_S on apoptosis [49,52]. Increasing studies have also been conducted to explore the role of H_2_S in ferroptosis and necroptosis [53,54], however whether H_2_S affects other forms of cell death needs to be further investigated.

Oxidative stress occurs as a consequence of the imbalance between the production and elimination of ROS [55]. The role of H_2_S in regulating oxidative stress by influencing the production of ROS and antioxidants has been explored in some detail [56,57,58]. The antioxidant effect of H_2_S involves various mechanisms, including directly quenching free radicals (in which H_2_S serves as a chemical reductant) and scavenging free radicals either by upregulating the levels of nonenzymatic antioxidants such as reduced glutathione and classic thioredoxin or affecting a suite of cellular antioxidant enzymes, such as superoxide dismutase, catalase and glutathione peroxidase. Moreover, inhibiting the generation of mitochondrial free radicals also participates in the protection provided by H_2_S to cells against oxidative stress [56,57,58]. However, several investigations have indicated that H_2_S also has pro-oxidative properties and this aspect has been overviewed in a comprehensive review [59].

Taking advantage of the development of chemical and genetic H_2_S research tools and the recognition of the nature of being a molecular signaling mediator of H_2_S, the complex roles of H_2_S in a plethora of acute/chronic inflammatory conditions, such as pulmonary diseases (see the detailed discussion in Section 4), gastrointestinal disorders, sepsis (systematic inflammation) and arthritis (local inflammation), have been substantially investigated and become increasingly clear [27,28,60]. However, the precise role of H_2_S in the inflammatory process is still ambiguous and yet to be fully understood as both pro-inflammatory and anti-inflammatory properties of H_2_S have been proposed [28,60,61].

## 4. Role of H_2_S in Pulmonary Diseases

Most of the H_2_S metabolism pathways participate in the biosynthesis and catabolism of H_2_S in the respiratory system. In addition, perturbations of these pathways have been observed in several pulmonary diseases. Moreover, H_2_S acts as a key contributor in the maintenance of many respiratory functions in addition to a signaling mediator bridging cell death, oxidative stress and inflammation, which are three significant mechanisms involved in the onset and progression of pulmonary disorders. These facts highlight the importance of having a comprehensive understanding of the significant roles played by H_2_S in the respiratory system [23,24,25]. In the past few decades, a great effort has been made to investigate the effects of H_2_S on the pathogenesis of many pulmonary diseases, including ALI/ARDS, pneumonia, asthma, COPD and lung cancer.

### 4.1. H_2_S and Acute Lung Injury/Acute Respiratory Distress Syndrome

ALI that manifests itself clinically as ARDS is a common pathological condition characterized by acute hypoxemic respiratory failure and bilateral pulmonary edema. Consequently, the morbidity and mortality of ALI/ARDS are both very high [1,62,63]. Many direct (pulmonary) and indirect (extrapulmonary) insults can lead to ALI/ARDS. Pneumonia and sepsis/endotoxemia, respectively, rank first as the most common direct and indirect risk factors of ALI/ARDS [63]. It has been suggested that H_2_S is extensively involved in the pathophysiology of ALI/ARDS induced by different insults.

#### 4.1.1. H_2_S and Sepsis/Endotoxemia-Induced Acute Lung Injury

**Figure 2:**
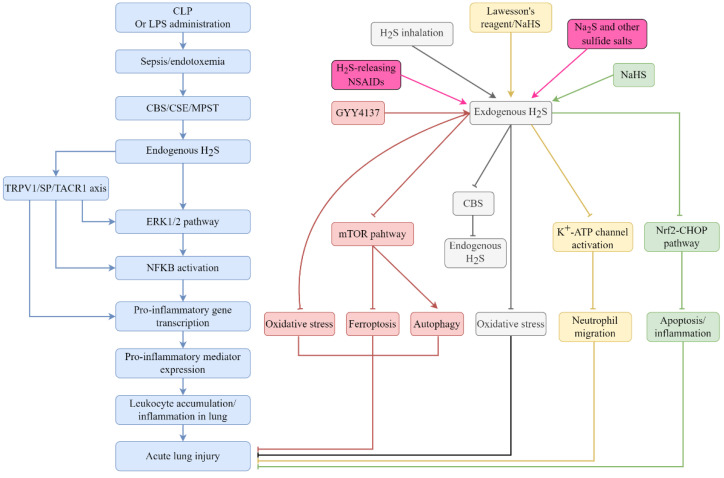
Overview of the role of H_2_S in sepsis/endotoxemia-induced acute lung injury. The induction of sepsis or endotoxemia in animals leads to an increase in the expressions of CBS, CSE and MPST, and further results in an upregulation in host-derived H_2_S. Subsequently, endogenous H_2_S imbalance dysregulates several inflammatory pathways and further causes remote lung injury. In contrast, the supplement of exogenous H_2_S may be therapeutically advantageous as it protects animals against lung injury in sepsis. Abbreviations: cecal ligation and puncture (CLP), lipopolysaccharide (LPS), transient receptor potential vanilloid type 1 (TRPV1), substance P (SP), tachykinin receptor 1 (TACR1), extracellular signal-related kinases (ERK), mechanistic target of rapamycin (mTOR), nuclear factor erythroid 2—related factor 2 (Nrf2), C/EBP homologous protein (CHOP).

Sepsis is a life-threatening organ dysfunction that arises as a consequence of the host response failure to control invading pathogens and their toxins. Sepsis affects around 30 million individuals worldwide and its incidence continues to rise [64,65]. The lungs are the most vulnerable organs during the development of sepsis/endotoxemia; hence, sepsis/endotoxemia frequently causes many alterations in the lung tissues [66,67]. The roles of H_2_S as a signaling mediator and a potential therapeutic target in ALI caused by sepsis/endotoxemia have been widely investigated [27,28] and are briefly summarized in Figure 2.

In a mouse model of endotoxemia, intraperitoneal injection of lipopolysaccharide (LPS) led to higher CSE expression and H_2_S biosynthesis, accompanied with increased systematic inflammation and severe lung injury [68]. Inhibiting the catalytic activity of CSE and therefore decreasing the production of host-derived H_2_S by DL-Propargylglycine (PAG) treatment showed a protective effect on endotoxemia-induced lung injury, as evidenced by lower activity of myeloperoxidase and less structural destructions in lung tissue [68]. The pro-inflammatory actions of endogenous H_2_S in lung injury were also observed in mice with cecal ligation and puncture (CLP)-induced sepsis [69]. Thereafter, many studies have been conducted to further investigate the involvement of endogenous H_2_S in sepsis-induced organ dysfunction and elucidate the potential mechanism. Mechanistically, the elevated H_2_S biosynthesis triggered by CLP was linked to the activation of NF-κB and the following rise in the productions of cytokines, chemokines and adhesion molecules in mice with sepsis. As a result, the rolling and adherence of leukocytes were significantly increased, leading to a serious injury in the lung tissues [70,71]. Subsequently, it has been shown that the pro-inflammatory effects of endogenous H_2_S on lung injury caused by sepsis in mice were achieved by activating the extracellular signal-related kinases (ERK)/NF-κB pathway [72]. A series of investigations have indicated that the transient receptor potential vanilloid type 1 (TRPV1)/substance P/tachykinin receptor 1 axis plays a significant role in the activation of the ERK/NF-κB pathway and ensuing lung inflammation and injury mediated by elevated H_2_S following sepsis [73,74,75]. It has been suggested that elevated host-derived H_2_S induced by CLP led to the activation of TRPV1, thereby increasing the productions of cyclooxygenase-2 and prostaglandin E metabolite and ultimately causing inflammation and injury in lung tissues in mice [76]. Moreover, in a mouse model of CLP-induced sepsis, the treatment of small interfering RNA targeting CSE significantly mitigated sepsis-associated lung inflammation and injury, further confirming the pro-inflammatory effects of endogenous H_2_S in sepsis [77].

On the contrary, some investigations also suggested a protective effect of H_2_S in sepsis-induced ALI. In a mouse model of CLP-induced sepsis, reducing H_2_S biosynthesis via PAG treatment caused significant aggravation of lung injury, whereas the administration of exogenous H_2_S obviously ameliorated sepsis-induced alterations in lung tissues [78]. Although the rolling and adhesion of leukocytes were consistently reduced following the reduction of H_2_S biosynthesis [70,71,78], the effects of inhibiting host-derived H_2_S on sepsis-induced lung injury were opposing. Different health statuses, ages and genders of the animals, different commercial sources of PAG and different severities of sepsis may explain the discrepancy [78]. Several recent studies also pointed to the protective effects of H_2_S in CLP-induced lung injury as the exogenous H_2_S supplement suppressed apoptosis and ferroptosis in lung tissues and ultimately abrogated the development of lung injury in mice with CLP-induced sepsis [53,79]. Recently, increased CBS expression in the lung tissues, accompanied by excessive inflammatory and oxidative responses, was observed in mice receiving LPS instillation. Interestingly, H_2_S inhalation showed a negative feedback effect on CBS expression in the lung tissues, subsequently leading to a significant downregulation in the expressions of stress-related protein and activated MAP kinase and the formation of ROS, thereby ultimately mitigating lung inflammation and injury caused by LPS instillation [80].

#### 4.1.2. H_2_S and Acute Pancreatitis-Induced Acute Lung Injury

Acute pancreatitis, a common pancreatic disorder characterized by a local and systemic inflammatory response, often causes multiple organ dysfunction [81]. The incidence of acute pancreatitis keeps increasing worldwide and the global morbidity and mortality are still very high [82]. The lungs are one of the most easily affected organs aside from the pancreas during the onset, development and progression of acute pancreatitis [83]. As H_2_S imbalance has been implicated in acute pancreatitis, the actions played by H_2_S in acute pancreatitis-induced ALI are becoming increasingly clear [24,28].

In a mouse model of acute pancreatitis induced by caerulein administration, the disease induction led to the activation of the CSE/H_2_S pathway in pancreas tissues, which significantly amplified inflammation and injury in the pancreas and lungs [84]. Treatment with PAG robustly suppressed the biosynthesis of H_2_S, which in turn led to protection against ALI following acute pancreatitis [84]. Subsequently, it was reported that the detrimental effects of H_2_S on acute pancreatitis were mediated by chemokines, as the treatment with PAG to inhibit the catalytic activity of CSE not only reduced the expressions of pro-inflammatory chemokines in the pancreas and lungs but also attenuated the severity of acute pancreatitis-induced alterations in lung tissues [85]. A recent study also suggested that the elevated H_2_S biosynthesis catalyzed by CSE exacerbated inflammation and injury in lung tissues caused by acute pancreatitis [86]. In line with studies establishing an acute pancreatitis model in mice by caerulein injection, in a rat model with sodium deoxycholate injection-induced acute pancreatitis, the activated CSE/H_2_S pathway was linked to the significant aggravation in inflammation and injury in lung tissues [87].

H_2_S may also provide significant protection against acute pancreatitis-induced ALI as treating the mice with H_2_S-releasing diclofenac derivative (ACS15) obviously attenuated lung inflammation and injury following acute pancreatitis [88]. The slow release of H_2_S from ACS15 may explain the protective actions of H_2_S in acute pancreatitis-induced ALI. Another study indicated that a low dose of sodium hydrosulfide (NaHS) protected mice against acute pancreatitis-induced ALI and the protective effects of H_2_S were achieved by inhibiting caerulein injection-induced inflammation via downregulating the expressions of pro-inflammatory chemokines and adhesion molecules [89].

#### 4.1.3. H_2_S and Burn/Inhalation-Induced Acute Lung Injury

It has been shown that the mRNA levels of CSE and endogenous H_2_S synthesis activity were markedly upregulated, and the lung tissues were obviously damaged in mice with severe burn injuries. The administration of PAG significantly attenuated burn injury-induced systematic inflammation and remote lung injury, while the treatment of NaHS further exacerbated these alterations [90]. In contrast, in a mouse model of ALI induced by a combination of burn and smoke inhalation, the exogenous H_2_S supplement primed the anti-inflammatory and antioxidant pathways, which significantly mitigated lung injury and reduced mortality [91]. The discrepancy between these studies is probably due to different animal models and H_2_S supplements used (NaHS vs. H_2_S parenteral formulation). A recent study showed that the endogenous production of H_2_S obviously increased in mice at the early stage of burn injury (within 24 h) but turned to the baseline level at the late stage (after 3 days). Intriguingly, the treatments with the H_2_S biosynthesis inhibitor (aminooxyacetic acid (AOAA)) and the H_2_S donor (AP39) provided similar protective effects in burn injury-induced systematic inflammation and lung injury in a mouse model of burn injury, as both AOAA and AP39 treatment mitigated burn-induced increases in lung neutrophil accumulation (activity of lung myeloperoxidase) and oxidative stress (level of lung malondialdehyde), as well as burn-induced elevation in the systematic inflammatory response (levels of plasma pro-inflammatory cytokines) [92]. Slow H_2_S-releasing donors cause an inhibition of endogenous H_2_S formation, possibly by a negative feedback mechanism, which may explain the anti-inflammatory actions of slow H_2_S-releasing compounds [28]. Therefore, the anti-inflammatory actions of AP39 (a slow H_2_S-releasing donor) probably contribute to its protective effects on burn-induced ALI. The antioxidant and cytoprotective effects of upregulating the level of mitochondrial H_2_S on inflammation and organ stress have been extensively confirmed [93]. Thus, the beneficial effect of AP39 was also assumed to be its nature of being a mitochondria-targeted H_2_S donor, which might preferably accumulate in the mitochondria and selectively increase the concentration of mitochondrial H_2_S without significantly upregulating the systematic H_2_S concentration [92].

#### 4.1.4. H_2_S and Ventilator-Induced Acute Lung Injury

Mechanical ventilation can cause several lung alterations in mice, including significant inflammation and injury. In contrast, continuous H_2_S inhalation (80 parts per million) during mechanical ventilation inhibited ventilation-induced pro-inflammatory and apoptotic responses, thereby effectively protecting mice against ventilator-induced lung injury (VILI) [94]. A subsequent study indicated that the protective effects of H_2_S inhalation in VILI in mice were probably achieved by inhibiting the expressions of genes that are associated with oxidative stress and inflammation while increasing that of genes related to anti-apoptosis and anti-inflammation and genes controlling the vascular permeability [95]. Thereafter, this research group has pointed to the involvement of the PI3K/AKT pathway in the protective effect of H_2_S inhalation in VILI in mice. Specifically, the PI3K/AKT pathway was activated by H_2_S inhalation, which further attenuated mechanical ventilation-induced inflammation and oxidative stress [96]. More recently, H_2_S inhalation has been shown to protect rats against VILI, as evidenced by reduced levels of inflammatory response, histopathological damage, pulmonary edema and permeability, as well as oxidative stress. Moreover, the protective effect of H_2_S inhalation was achieved by impeding the activation of the PERK/eIF2α/ATF4/GADD34 pathway and the NF-κB/MAPK pathway, which further led to an obvious decline in apoptosis, autophagy and endoplasmic reticulum stress [97]. The exogenous H_2_S supplement also provided protection to rats against VILI as a continuous NaHS infusion reduced lung inflammation and improved oxygenation, which further protected rats against VILI [98]. Another study indicated that a systemic supplement of exogenous H_2_S by intravascular injection with Na_2_S is an effective therapeutic approach to prevent VILI, whereas a continuous inhalation of H_2_S (60 parts per million) during mechanical ventilation accelerated the development of VILI in mice [99]. The discrepancy regarding the effects of H_2_S inhalation in VILI among studies is probably due to different animal models and different doses of H_2_S used.

### 4.2. H_2_S and Pneumonia

Pneumonia represents a common acute respiratory infection with high morbidity and mortality [2]. A variety of pathogens, including bacteria, respiratory viruses and fungi, can cause pneumonia [2,100]. Aberrant H_2_S metabolism has been implicated in several types of pneumonia.

A recent study has pointed to the involvement of H_2_S in the pathogenesis of tuberculosis [101]. In a landmark study, it was shown that the levels of CSE and MPST were higher in human tuberculous lung tissues than in healthy lung tissues. Moreover, CSE-derived H_2_S profoundly affected the immune response of mice by altering the central carbon metabolism, thereby ultimately exacerbating tuberculosis [102]. Consistently, mice infected by *Mycobacterium tuberculosis* (*Mtb*) by the aerosol route had higher levels of CBS, CSE and MPST in lung tissues compared with normal mice [102]. Taking advantage of using GYY3147 and PAG as well as establishing pulmonary tuberculosis in mice genetically deficient in the CSE gene, researchers also demonstrated that elevated H_2_S catalyzed by CSE not only enabled the *Mtb* to survive and grow in macrophages during infection but also dysregulated the host immune response to *Mtb* infection and impaired central carbon catabolism in mice, thereby enhancing virulence and infection of *Mtb* and eventually promoting the progression of pulmonary tuberculosis [102]. Similarly, elevated host-derived H_2_S catalyzed by CBS was also shown to participate in the pathogenesis of pulmonary tuberculosis induced by *Mtb* infection in mice [103]. It was reported that CBS-catalyzed H_2_S can modulate the growth, energy homeostasis (bioenergetics and ATP production) and central metabolism of *Mtb.* Moreover, it was also shown that genetic CBS deletion enhanced the survival probability and decreased the organ burden, and the treatment with the CBS inhibitor reduced the bacterial load in mice following *Mtb* infection [103].

H_2_S imbalance has also been linked to the pathogenesis of viral pneumonia. Endogenous production of H_2_S has been reported to be decreased in airway epithelial cells infected by the respiratory syncytial virus (RSV) as a consequence of lower expression of CSE (mRNA and protein) and increased degradation of H_2_S [104]. The inhibition of CSE with PAG obviously enhanced the replication of RSV and the secretion of pro-inflammatory chemokines, whereas the supplement of exogenous H_2_S with GYY4137 showed a significant suppression effect on these processes by inhibiting the nuclear translocation of NF-κB and interferon regulatory factor 3, which are two crucial transcription factors involved in infection [104]. These findings led them to further investigate the role of H_2_S metabolism in mice with RSV infection [105]. In this study, the levels of both CBS and CSE were lower in lung tissues in mice with RSV infection than in normal mice [105]. The exogenous H_2_S supplement by GYY4137 intranasal delivery significantly provided protection to mice against RSV infection, whereas mice genetically deficient in CSE exhibited more serious disease, as evidenced by increased airway reactivity, increased viral replication in lung tissues and increased lung inflammation compared with wild-type mice [105]. Several clinical studies have also shed light on endogenous H_2_S in severe acute respiratory syndrome coronavirus 2 (SARS-CoV-2) infection (coronavirus disease 2019, COVID-19) [106]. Patients with COVID-19 had lower levels of plasma H_2_S than healthy controls, however lower levels of plasma H_2_S indicated a higher survival probability [107]. In contrast, the levels of serum H_2_S were also found to be positively correlated with the final survival probability of COVID-19 [108]. The reason for the discrepancy in terms of the correlation between endogenous H_2_S and the severity and outcome of COVID-19 is not yet clear. Regarding this controversy, many investigations have concluded that H_2_S plays a key role in preventing SARS-CoV-2 infection as it affects the interaction between host cells and SARS-CoV-2, thereby blocking the cellular entry of SARS-CoV-2, as discussed in a comprehensive review [109].

### 4.3. H_2_S and Chronic Obstructive Pulmonary Disease

COPD is a chronic airway inflammatory disease with persistent respiratory symptoms and progressive airflow obstruction [110]. On the global scale, it affects approximately 400 million people and causes more than 3 million deaths, making it a leading cause of global morbidity, mortality and healthcare use [111]. Increasing studies have demonstrated that endogenous H_2_S is not only involved in the pathogenesis of COPD, but it is also closed related to the severity of COPD [112].

Since cigarette smoking (CS) is the predominant causative factor of COPD [111,113], a CS exposure-induced COPD rodent model has been treated as a useful tool in COPD research [114]. Tang et al. showed that 4 months of CS exposure resulted in an upregulation of CSE expression and H_2_S biosynthesis and several COPD-associated abnormalities, including increased airway reactivity and lung injury characterized by epithelial damage, inflammation and emphysema in rats. Importantly, these COPD-associated abnormalities were linked to the abnormal activation of the CSE/H_2_S pathway as the supplement of exogenous H_2_S with NaHS ameliorated increased airway reactivity and lung injury, while inhibiting H_2_S biosynthesis by pharmacologically blocking CSE with PAG further aggravated these aforementioned abnormalities [115]. In contrast, the expressions of CBS and CSE were reported to be significantly decreased in the lung tissues in rats after chronic CS exposure (3 or 6 months), accompanied by an obvious drop in H_2_S endogenous production [116]. However, the administration of NaHS protected rats against CS exposure-induced cell death, oxidative stress and airway inflammation and remodeling, and as such, suppressed the development of emphysema and pulmonary hypertension [116]. The protective effects of exogenous H_2_S were achieved by activating the AKT pathway and consequently preventing the decrease of Nrf2 [116]. Consistently, a recent study indicated that the mRNA levels of CBS, CSE and MPST were downregulated in lung tissues in mice after CS exposure [117]. Apart from CS exposure, atmosphere particulate matter exposure also serves as an important etiological factor for the development of COPD [118]. The protein level of CSE was significantly decreased in the lung tissues in mice with particulate matter (PM) exposure-induced COPD [119]. In addition, the inhibition of H_2_S biosynthesis with PAG further exacerbated PM exposure-induced lung inflammation and emphysema in mice. In contrast, the supplement of exogenous H_2_S by NaHS administration significantly protected mice against PM exposure-induced airway abnormalities via accelerating ROS scavenging and inhibiting pyroptosis and apoptosis in the airway in an Nrf2-dependent manner [119].

In addition to animal studies, a great effort has been made by several research groups in investigating the profile of endogenous H_2_S in patients with COPD. Endogenous production of H_2_S has been shown to be significantly altered in COPD patients [120]. The levels of serum H_2_S in patients with stable COPD were higher than in those with acute exacerbation of COPD (AECOPD) and healthy controls [120]. In all individuals, smokers had lower levels of serum H_2_S than in nonsmokers [120]. Moreover, in cases with stable COPD, the levels of serum H_2_S were higher in patients with stage I airway obstruction compared to those with stage III airway obstruction [120]. In all patients, the levels of serum H_2_S showed a positive correlation with lung functions of patients [120]. While a negative correlation between the levels of serum H_2_S and the proportion of neutrophils in sputum was observed, the levels of serum H_2_S were positively correlated with the proportions of lymphocytes and macrophages in sputum [120]. This study indicated that endogenous H_2_S played a crucial role in airway obstruction development in COPD and the levels of serum H_2_S were associated with the activity and severity of COPD [120] and aroused great attention in exploring the levels of H_2_S in different samples in addition to serum in COPD. It has been shown that the levels of H_2_S in exhaled breath were similar in patients with stable COPD, AECOPD and healthy controls [121]. In this study, the levels of exhaled H_2_S were not significantly correlated with the proportions of different cell types in sputum, however, the levels of H_2_S in exhaled breath were found to be negatively correlated with the proportion of eosinophils in sputum in another study [121,122]. This discrepancy indicated that further research with larger population cohort is required as the populations of these two studies were relatively small (38 and 77, respectively) [121,122]. Interestingly, exhaled H_2_S levels were positively correlated with inspiratory capacity in COPD patients and lower levels of exhaled H_2_S were found in patients with eosinophilia and more frequency of acute exacerbations [122]. The profiles of H_2_S in sputum were also investigated in COPD. Specifically, COPD patients had higher levels of sputum H_2_S compared with healthy individuals and the levels of sputum H_2_S were higher in patients with AECOPD compared with those with stable COPD [123]. The pathway of H_2_S biosynthesis also differed in lung tissues in patients with COPD [124]. The levels of CSE protein and CBS mRNA in lung tissues in COPD patients were lower than in healthy controls. However, the mRNA levels of CSE in lung tissues were higher in COPD patients compared with healthy individuals and the levels of lung H_2_S were similar between patients and healthy controls [124]. More recently, endogenous H_2_S was also linked to pulmonary vascular remodeling in COPD patients [125]. In addition, it has been shown that the production of polysulfide species in lung tissues of patients with COPD was lower than that of non-COPD patients [126]. Although the profile of H_2_S was not a focus of this study, considering the important role of polysulfide species in H_2_S metabolism, this research might also indirectly echo the potential actions played by H_2_S in COPD.

### 4.4. H_2_S and Asthma

Asthma is a common chronic inflammatory disorder of the airways [127]. As a heterogeneous clinical syndrome, asthma affects more than 300 million individuals globally and has a high ratio of death [128]. Increasing evidence has indicated that endogenous H_2_S presents itself as a crucial mediator in the pathogenesis of asthma [112].

Ovalbumin-induced asthma models in rodents have been widely used in investigating the pathogenesis of allergic asthma as asthma in these models mimics many aspects of asthma in humans [129]. As shown in the landmark study that firstly shed light on the role of H_2_S in asthma, researchers found that the levels of CSE in lung tissues (both mRNA and protein) in rats with ovalbumin-induced asthma were 42.6% and 63.4% lower than those in lung tissues from normal rats [130]. In addition, the catalytic activity of CSE was 78.3% lower in asthmatic rats than in control rats [130]. These significant declines in the expression and enzymatic activity of CSE led to an obvious decrease in the levels of endogenous H_2_S in lung tissues and serum (79.8% and 81.1%, respectively) [130]. Notably, the levels of endogenous H_2_S were found to be positively correlated with the lung function (peak expiratory flow) of rats, whereas a negative correlation between the level of H_2_S and the proportion of eosinophils and neutrophils in bronchoalveolar lavage fluid and asthma-associated lung alterations, such as inflammatory cells’ infiltration, collagen deposition, goblet cells’ proliferation and airway smooth-muscle hyperplasia, was observed as well [130]. Moreover, the administration of exogenous H_2_S by injecting NaHS obviously mitigated ovalbumin-induced alterations [130]. The involvement of the CSE/H_2_S pathway in asthma has been confirmed in further studies [131,132]. In these studies, the expression of CSE and endogenous generation of H_2_S dropped significantly in lung tissues in mice after ovalbumin challenge. Importantly, while many asthma-associated alterations, including increased airway responsiveness, airway inflammation and lung damage, were exacerbated in mice genetically deficient in CSE, theses abnormalities induced by ovalbumin challenge were obviously attenuated by the supplement of exogenous H_2_S via NaHS injection [131,132]. Notably, it has been demonstrated that the protective effects of exogenous H_2_S on asthma are achieved by attenuating airway hyperactivity and pulmonary fibrosis by inhibiting OVA-induced activation of mast cells in lung tissues in mice, as NaHS inhalation was shown to reverse OVA-induced degranulation and fibrogenic cytokines’ generation in mast cells [133].

As asthma affects all age groups of humans, some studies with an aim to unravel the profiles and the roles of endogenous H_2_S in adult and pediatric asthma patients have been conducted. In agreement with what has been found in rodents, the levels of H_2_S in serum and exhaled breath have been found to be apparently decreased in asthma individuals [134,135,136]. CBS-derived H_2_S has been shown to profoundly affect the function of airway smooth-muscle cells in asthma patients. In addition, exogenous H_2_S has further been shown to inhibit the activation of the ERK1/2 and p 38 pathways, which further inhibits the proliferation and cytokine release in airway smooth-muscle cells [137]. However, some investigations showed an increase in the levels of H_2_S in induced sputum samples from asthma patients. Moreover, in comparison with stable patients, uncontrolled patients and patients with acute exacerbation had even higher levels of sputum H_2_S [138,139]. Given that microbiota-produced H_2_S is also a key source of endogenous H_2_S, changes in H_2_S-producing microorganisms in the oral cavity could possibly be one reason why the profiles of H_2_S in induced sputum samples and in other samples are distinctively different [140]. It should be noted that a research group also indicated that the levels of serum H_2_S increased in asthma patients [138].

### 4.5. H_2_S and Lung Cancer

Lung cancer is the second most frequently diagnosed cancer worldwide (2.2 million newly diagnosed cases in 2020). In addition, as a highly lethal disease, lung cancer is responsible for 18.0% of cancer deaths, making it a prominent source of mortality attributable to cancer [141]. It has been proposed that the expressions and enzymatic activities of H_2_S-synthesizing enzymes are increased in many forms of human malignancy. More importantly, aberrant H_2_S metabolism acts as a key contributor to the onset and progression of several tumors. Therefore, H_2_S has been recently accepted as an oncogenic mediator in many cancers [142].

The protein levels of CBS, CSE and MPST were higher in lung adenocarcinoma tissues compared with adjacent normal lung tissues, accomplished by an increase in H_2_S biosynthesis. Similarly, lung adenocarcinoma cells, in comparison to normal lung epithelial cells, expressed higher levels of CBS, CSE and MPST and produced higher levels of H_2_S [143]. Of note, researchers indicated that the increased H_2_S biosynthesis gave birth to chemotherapy resistance in lung adenocarcinoma. The effect of H_2_S involves its ability in enhancing mitochondrial DNA repair (via enhancing the formation of mitochondrial DNA repair complex) and controlling mitochondrial biogenesis in lung adenocarcinoma [143]. Moreover, the administration of AOAA or silencing of CBS suppressed the endogenous production of H_2_S, further leading to a drop in the viability of camptothecin (DNA topoisomerase I inhibitor)-treated lung adenocarcinoma cells, thereby eventually increasing the sensitivity of lung adenocarcinoma cells to chemotherapeutic drugs [143]. More recently, the upregulated expression and catalytic activities of CBS, CSE and MPST in lung cancers were further confirmed [144]. In this study, the increased generation of endogenous H_2_S favored the migration and invasion, epithelial-to-mesenchymal transition and tumor angiogenesis in lung cancer by modulating the activation of hypoxia-inducible factor-1α [144]. Specifically, in vitro, inhibiting host-derived H_2_S by treating the cells with small interfering RNA targeting CSE or CBS suppressed the proliferation and metastasis potential of tumor cells; in vivo, the tumor angiogenesis and lung cancer cells’ growth were significantly suppressed by treating the mice with AOAA and PAG [144]. In contrast, in cisplatin-resistance lung cancer cells, the expression of CBS and the generation of H_2_S were lower than in normal lung cancer cells. Supplement of exogenous H_2_S not only inhibited the cell viability and increased the chemosensitivity of cisplatin-resistant lung cancer cells but also caused apoptosis, cell cycle arrest and suppressed migration and invasion of these cells. These effects of H_2_S were achieved by activating p53, increasing the expressions of p21, caspase-3, Bax and matrix metalloproteinase-2 and reducing the level of Bcl-xL. These findings indicate that a supplement of exogenous H_2_S is probably a promising therapeutic approach for lung cancer with cisplatin resistance [145].

### 4.6. H_2_S and Other Pulmonary Diseases

A growing number of studies have pointed to the potential role of abnormal H_2_S metabolism in many other pulmonary diseases, including idiopathic pulmonary fibrosis, cystic fibrosis, sleep apnea syndrome and pulmonary hypertension [23,24,25].

Idiopathic pulmonary fibrosis, a chronic and progressive lung disease, is characterized by progressive lung scarring [146]. As the most common form of interstitial lung illness, globally, idiopathic pulmonary fibrosis affects approximately 3 million individuals [146]. Dysregulated H_2_S metabolism has been observed in pulmonary fibrosis. In a rat model of pulmonary fibrosis, intratracheal instillation of bleomycin led to obvious alterations in the host CSE/H_2_S pathway. Decreased activity of lung CSE and lower levels of plasma H_2_S were found in rats with pulmonary fibrosis compared with normal rats. Intraperitoneal injection of NaHS suppressed oxidative stress and structural alterations in lung tissues induced by bleomycin instillation. These findings indicate that a deficiency in endogenous H_2_S production played a significant role in the pathogenesis of pulmonary fibrosis [147]. Consistently, it has been shown that intraperitoneal injection of NaHS significantly mitigated bleomycin-induced pulmonary fibrosis in rats, and the protective effects of exogenous H_2_S were attributed to its ability in inhibiting the expression of NF-κB and regulating the Th1/Th2 balance [148]. Similarly, the levels of H_2_S in plasma and in lung tissues were both obviously lower in rats with passive smoking-induced pulmonary fibrosis than in control rats. Smoking-induced oxidative stress and architectural abnormalities in the lung tissues were attenuated by the treatment of NaHS [149]. In this study, the protective effects of exogenous H_2_S were linked to its ability in inhibiting the activation of lung mitogen-activated protein kinases and NF-κB as well as promoting the transcription activity of lung Nrf2 [149].

Cystic fibrosis is a multisystem disease dominated by pulmonary and digestive manifestations and classical cystic fibrosis is characterized by chronic respiratory infection and inflammation and progressive respiratory decline [150]. As a rare genetic disease, cystic fibrosis affects about 100,000 individuals globally [151]. A small-scale clinical study indicated that aberrant H_2_S metabolism is also implicated in cystic fibrosis [152]. Higher levels of sputum H_2_S have been shown to be associated with lower sputum generation and decreased hospitalization and oxygen supplement treatment requirement in pediatric cystic fibrosis patients [152]. This study had several limitations, including a lack of healthy controls, which led to a cross-sectional nature, and the analyses were only performed in a cohort with a small population (only 22 pediatric patients were recruited).

Sleep apnea syndrome (SAS) is a prevalent respiratory sleep disorder characterized by repetitive apnea, chronic intermittent hypoxia (CIH) and hypercapnia [153,154]. On a global scale, the incidences of SAS in adults and children are both very high [155,156]. H_2_S imbalance has been implicated in SAS. Given that CIH serves as a key contributor to SAS pathogenesis, CIH-challenged rodents are widely used in SAS-related research. In comparison to normal rats, rats experienced a decrease in the activity and the expression of CSE in the mesenteric tissues after the induction of CIH. As a result, the endogenous generation of H_2_S was lower in these rats [157]. Similarly, the induction of CIH inhibited the expression of CSE, thereby resulting in a significant decrease in the endogenous production of H_2_S and attendant elevated oxidative stress, apoptosis and endoplasmic reticulum stress in the left ventricular myocardium in rats. However, the treatment of NaHS protected rats against CIH-induced myocardial injury as these alterations were obviously mitigated [158]. On the contrary, increased biosynthesis of H_2_S was also observed in the carotid body of rodents after the induction of CIH [159,160].

Pulmonary hypertension is a term that embraces many diseases characterized by unusually elevated high pressures in the pulmonary circulation. Pulmonary hypertension affects around 1% of the global population [161,162]. It has been reported that hypoxia exposure significantly decreases the gene expression of CSE, the enzymatic activity of CSE and the levels of H_2_S in lung tissues in rats, which further caused an increase in pulmonary arterial pressure and structural remodeling in pulmonary vessels and ultimately resulted in pulmonary hypertension. However, these hypoxia exposure-induced alterations were reversed by the exogenous H_2_S supplement [163]. Similarly, the CSE/H_2_S pathway was also found to be deficient during the development of pulmonary arterial endothelial cell injury-induced pulmonary hypertension in rats. Exogenous H_2_S supply via NaHS injection provided protection to rats against pulmonary hypertension by suppressing inflammation and vascular remodeling in lung tissues as well as reducing pulmonary arterial pressure [164]. Notably, these beneficial effects of exogenous H_2_S were attributed to its ability in inhibiting the transcription activity of NF-κB by inhibiting the release and nucleus translocation of NF-κB p65/p50 [164]. In addition, the protein levels of CBS, CSE and MPST in addition to the biosynthesis of H_2_S were found to be downregulated in small pulmonary arteries in rats with high pulmonary blood flow-induced pulmonary hypertension [165].

## 5. Conclusions

**Figure 3:**
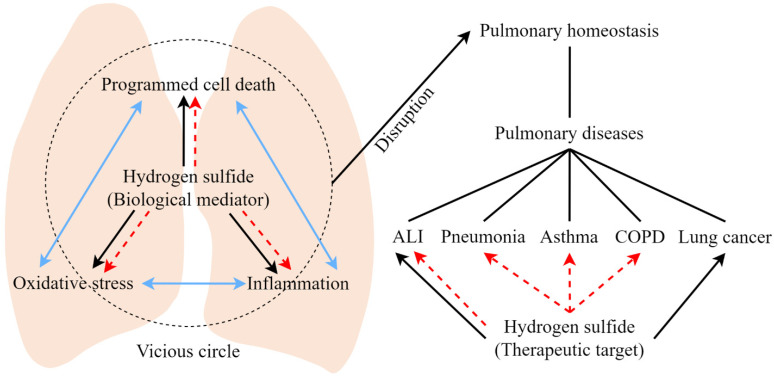
The schematic overview of the roles played by H_2_S as a biological mediator and a therapeutic target in pulmonary diseases. In living organisms, in response to pathological insults, programmed cell death, oxidative stress and inflammation can lead to each other (blue double-headed arrow), which triggers a local auto-amplification loop (black dashed circle). Under pathological conditions, H_2_S can either promote (black solid arrow) or inhibit (red dashed arrow) programmed cell death, oxidative stress and inflammation. As programmed cell death, oxidative stress and inflammation are three important biological processes extensively involved in maintaining the homeostasis of the respiratory system, H_2_S, functioning as a significant biological mediator, has been linked to the pathogenesis of several pulmonary diseases. Moreover, H_2_S has also been demonstrated as a potential therapeutic target in many pulmonary diseases.

Pulmonary diseases, including acute and chronic respiratory disorders, pose a heavy burden and threat to human health. It is becoming increasingly clear that programmed cell death, oxidative stress and inflammation are three significant mechanisms that underlie the pathophysiology of various pulmonary diseases. In addition, accumulating evidence has linked H_2_S to these aforementioned biological processes. As such, H_2_S has been proposed to be extensively involved in a wide panel of pulmonary diseases, including ALI induced by various insults, pneumonia, asthma, COPD, lung cancer, idiopathic pulmonary fibrosis, cystic fibrosis, sleep apnea syndrome and pulmonary hypertension. The recognition of H_2_S as a key contributor to the onset, development and progression of pulmonary diseases has led to the development of novel therapeutic approaches targeting H_2_S metabolism for pulmonary diseases (Figure 3 and Table 3). More investigations are, no doubt, needed to define the precise involvement of H_2_S in pulmonary disorders. Nonetheless, as a result of the recently generated knowledge in this area, from our laboratory and others, we can conclude that it is worth putting more effort into further investigation of the precise mechanism by which H_2_S serves as a gaseous mediator and therapeutic target for pulmonary diseases. This approach will facilitate the translation of this knowledge into clinical practice.

## Figures and Tables

**Table 1:** A brief summary of common H_2_S-producing enzyme inhibitors.

Name	Target	IC_50_ (μM)	Trait
Aminooxyacetic acid	CSE/CBS	~1.1 and ~8.5, respectively	Nonselective
DL-Propargylglycine	CSE	40	Selective, irreversible
D-Penicillamine	CSE	270	Selective ^a^
β-Cyanoalanine	CSE/CBS	14, Not applicable for CBS ^b^	Nonselective, reversible
L-aminoethoxyvinylglycine	CSE	1	Selective
Trifluoroalanine	CBS/CSE	66 and 289, respectively	Nonselective, reversible
Oxothiazolidine derivative	CSE	31.6	Selective, reversible
CH0004	CBS	1	Selective
HMPSNE	MPST	~2–30	Selective

Abbreviations: Hydrogen sulfide (H_2_S), cystathionine β-synthase (CBS), cystathionine γ-lyase (CSE), 3-mercaptopyruvate sulfurtransferase (MPST), half-maximal inhibitory concentration (IC_50_). ^a^ IC_50_ for CBS is 8.5 mM, indicating that D-Penicillamine is approximately 30 times more selective against CSE. ^b^ The inhibitory effect of β-Cyanoalanine on CBS only occurs at concentrations exceeding 1 mM.

**Table 2:** A brief summary of common H_2_S donor compounds.

Category	H_2_S-Releasing Speed	Examples
Inorganic H_2_S donor	Fast	NaHS, Na_2_S and CaS
Organic H_2_S donor	Fast/Slow	Lawesson’s reagent (Fast) and GYY4137 (Slow)
H_2_S-releasing NSAIDs	Slow	ACS15
Mitochondria-targeted H_2_S donor	Slow	AP39 and AP123
ROI-activated H_2_S donor	Slow	Peroxythiocarbamate
Thiol-activated H_2_S donors	Medium–fast	NSHD
Natural H_2_S donors	Slow	Diallyl trisulfide

Abbreviations: Sodium hydrosulfide (NaHS), sodium sulfide (Na_2_S), calcium sulfide (CaS), morpholin-4-ium-4-methoxyphenyl phosphonodithioate (GYY4137), non-steroidal anti-inflammatory drugs (NSAIDs), *N*-(benzoylthio) benzamide (NSHD), H_2_S-releasing diclofenac derivative (ACS15), anethole dithiolethione (AP39), hydroxythiobenzamide (AP123), reactive oxygen intermediates (ROI).

**Table 3:** A brief summary of the major role of H_2_S and H_2_S-based therapy in pulmonary diseases.

Pulmonary Diseases	Role of H_2_S (Enzymes Involved)	H_2_S-Based Therapy	Reference
Acute lung injury attributed to			
- Sepsis/endotoxemia	Mainly detrimental (CSE/CBS)	Administration of PAG; Genetic silencing or deletion of CSE; Supply of NaHS or Lawesson’s reagent; Inhalation of H_2_S	[53,68,69,70,71,72,73,74,75,76,77,78,79,80]
- Acute pancreatitis	Mainly detrimental (CSE)	Administration of PAG; Supply of ACS15 or NaHS	[84,85,88,89]
- Burn/inhalation	Both detrimental and protective (CSE)	Administration of PAG or AOAA; Supply of AP39 or H_2_S parenteral formulation	[90,91,92]
Ventilation	Mainly protective	Inhalation of H_2_S; Supply of NaHS or Na_2_S	[94,95,96,98,99]
Pneumonia attributed to			
*- Mtb* infection	Mainly detrimental (CSE/CBS) ^a^	Administration of PAG; Genetic deletion of CSE or CBS	[102,103]
- RSV infection	Mainly protective (CSE/CBS)	Supply of GYY4137	[104,105]
- SARS-CoV-2 infection	Both detrimental and protective ^a^	NA	NA
Chronic obstructive pulmonary disease	Mainly protective (CSE/CBS) ^a^	Supply of NaHS	[115,116,119]
Asthma	Mainly protective (CSE) ^a^	Supply of NaHS	[130,131,132,133]
Lung cancer	Mainly detrimental (CSE/CBS) ^a^	Administration of PAG or AOAA; Genetic silencing of CSE or CBS	[143,144,145]
Idiopathic pulmonary fibrosis	Mainly protective (CSE)	Supply of NaHS	[148,149]
Cystic fibrosis	Probably protective ^a^	NA	NA
Sleep apnea syndrome	Mainly protective	Supply of NaHS	[158]
Pulmonary hypertension	Mainly protective (CSE)	Supply of NaHS	[164]

Abbreviations: Not applicable (NA). ^a^ Human data are available.
